# Sinus bradycardia as a rare adverse event in patients receiving cyclosporine A after allogeneic hematopoietic stem cell transplantation.

**DOI:** 10.46989/001c.94362

**Published:** 2024-03-04

**Authors:** Magdalena Karasek, Maciej Majcherek, Bartłomiej Kuszczak, Agnieszka Szeremet, Olga Chyrko, Tomasz Wróbel, Anna Czyż

**Affiliations:** 1 Department of Haematology, Blood Neoplasms and Bone Marrow Transplantation Wroclaw Medical University; 2 Department of Haematology, Blood Neoplasms and Bone Marrow Transplantation Wroclaw Medical University; 3 University of Wrocław

**Keywords:** allogeneic hematopoietic stem cell transplantation, immunosuppressive therapy, cyclosporine A, cardiological complications, bradycardia, CSA, alloHSCT

## Abstract

Cyclosporine A (CSA) is a commonly used immunosuppressive agent for the prophylaxis of graft-versus-host disease following allogeneic hematopoietic stem cell transplantation (alloHSCT). While tachycardia is a known adverse effect of CSA, bradycardia remains a phenomenon rarely described in the literature.

We conducted a retrospective evaluation of the incidence of bradycardia in patients after alloHSCT treated with CSA between January 2020 and February 2023 at our center.

Out of 206 patients, sinus bradycardia following the administration of CSA was observed in 6 (2.9%), comprising 3 women and 3 men, with the median age of 55 years (range: 20-65). The underlying diseases were myeloid malignancies in 4 and aggressive lymphoma in 2 patients. The patients received grafts from a matched unrelated (n=5) or a haploidentical family donor (n=1) following various conditioning regimens. Coexisting cardiovascular disorders were found in 5 of the 6 patients. All patients experienced symptomatic bradycardia within 1-4 days (median 2 days) after CSA introduction, which persisted until CSA withdrawal. One patient required treatment with atropine. All patients continued their immunosuppressive therapy with tacrolimus, which was well-tolerated Our study indicates CSA as a causative factor of sinus bradycardia in a small percentage of alloHSCT patients receiving CSA as graft-versus host disease (GvHD) prophylaxis. Importantly, these patients did not experience any cardiac complications when switched to tacrolimus. Although further research on the effects of CSA on heart automatation is needed, our single-center experience can help prompt diagnosis and therapeutic intervention in daily clinical practice.

## INTRODUCTION

Cyclosporine A (CSA) was the first and contimues to be one of the most effective immunosuppressive drugs to date. CSA binds with cyclophilin, forming a complex that effectively inhibits calcineurin, thereby hindering the activation of the nuclear factor of activated T-cell. The immunosuppressive and anti-inflammatory effects of CSA are due to a decrease in interleukin-2 transcription and suppression of activation of effector T cells.^1,2^ This distinct mode of action has found extensive application across various medical disciplines over the years, including dermatology, rheumatology, and transplantation. In hematology, cyclosporine was the first calcineurin inhibitor (CNI) used for graft-versus-host disease (GvHD) prophylaxis in combination with a short course of methotrexate (MTX). Nowadays, CNIs, encompassing both cyclosporine and tacrolimus, are still the basis of immunosuppression protocols, both in the “gold-standard” one, i.e. CNI+MTX, as well as newer approaches based on the combination of CNI and mycophenolate mofetil (MMF) with post-transplant cyclophosphamide (PT-Cy). Despite substantial clinical experience, the initial days of CSA administration carry a high risk of adverse effects; therefore, gradual dose titration and intensified patient monitoring are required.^2^ The response to CSA can be unpredictable, due to its pharmacokinetic variability, which depends on various factors such as age, the function of vital organs, the type of organ transplant, and concomitant medications.^3^

Cyclosporine has a narrow therapeutic index and can result in various toxic effects. Serious clinical consequences are linked to subtherapeutic concentrations, leading to GvHD, and supratherapeutic concentrations, which may cause nephrotoxicity. However, adverse effects can even appear at therapeutic doses. These effects encompass hypertension, hyperglycemia, central nervous system (CNS) toxicities, a potential increased susceptibility to infections, and an increased risk of reactivation of latent viral infections.^3–6^

Despite the well-documented safety profile, it is worth noting that some rare but serious side effects can occur as a result of CSA treatment. One such rare toxicity, especially in the context of alloHSCT, is cardiac arrhythmia, particularly bradycardia. It should be noted that this side effect is rarely discussed in the available literature on the subject. To enhance the published experience, we conducted a retrospective review of patients who underwent alloHSCT at our center, and experienced bradycardia while on CSA-based GvHD prophylaxis during alloHSCT. We intended to determine the frequency of this complication, to describe the characteristics of the affected patients, the clinical course of bradycardia, and, finally, to search for a possible cause of CSA-induced cardiotoxicity.

## METHODS

This study is a retrospective analysis of all consecutive patients who underwent alloHSCT between 2020 and 2023 in the Department of Hematology, Blood Neoplasms and Bone Marrow Transplantation of Wroclaw University Hospital. Out of all patients, we identified those who presented bradycardia during their transplant hospitalization while on CSA treatment. Basic information was collected from patients’ medical records, including gender, age, type of hematologic disease, indication for alloHSCT, history of treatment with cardiotoxic agents prior to transplant, comorbidities, and cardiovascular risk according to SCORE2 (Systematic Coronary Risk Estimation 2) and SCORE-OP2 (Systematic Coronary Risk Estimation 2-Older Persons), which are used in high cardiovascular risk populations, such as the Polish population. Details of the transplantation, such as the conditioning regimen, donor type, and GvHD prophylaxis protocol, were also recorded.

To thoroughly describe and analyze the bradycardia episodes, we included data on electrolyte levels (potassium [K+], calcium [Ca2+], magnesium [Mg2+]), glucose and CSA concentration at the onset of bradycardia, symptoms of bradycardia, electrocardiography (ECG) recordings, medications taken, acute neurological disorders, and active infections. Bradycardia was diagnosed based on ECG recordings as a heart rate below 60 beats per minute (BPM). According to the center’s standard protocols, the initial dose of CSA was 1.5 mg/kg every 12 hours in a slow six-hour infusion, aiming for a CSA blood concentration of 200 – 250 ng/mL during the first four weeks post-alloHSCT. At the time of posttransplant care, patients received intravenous infectious prophylaxis: levofloxacin (intravenously, 500 mg per day), acyclovir (intravenously, 250 mg every 8 hours) and fluconazole (intravenously, 200 mg twice a day) along with multi-electrolyte fluids. Electrolyte levels were monitored daily, and any imbalances were promptly corrected if necessary.

## RESULTS

### Patients’ characteristics

Among the 232 patients who underwent alloHSCT from 2020 to 2023, 6 (2.6%) developed bradycardia during CSA. This group comprised 3 females and 3 males, with a median age of 55 years (range: 20-65 years). Of these patients, 4 (66.6%) were diagnosed with myeloid malignancies, and the remaining 2 had aggressive lymphoma. Four of the patients received anthracycline-containing chemotherapy regimens before transplantation. Coexisting cardiovascular disorders were found in 5 of the 6 patients. The one without any cardiological comorbidities had a small to medium 10-year risk (2%) of developing cardiovascular diseases, either non-fatal or fatal, as estimated by the SCORE2. None of the patients analyzed were diagnosed with hypothyroidism or any other endocrine disorder.

A detailed breakdown of these characteristics is presented in Table 1.

**Table 1.** Characteristic of patients.

No.	Gender	Age (years)	Basic diagnosis	DRI	Cardiovascular disorders
1.	Male	65	PMF	Intermediate	Deep vein thrombosis
2.	Male	58	NHL-MCL	Very high	Hypertension, mitral and tricuspid regurgitation, left ventricular hypertrophy
3.	Female	52	AML	Intermediate	Asymptomatic anthracycline cardiomyopathy
4.	Female	53	NHL-MCL	Intermediate	None
5.	Female	20	ALL	High	Deep vein thrombosis
6.	Male	74	MDS	High	Two episodes of myocardial infarction and stroke

Abbreviations: PMF – primary myelofibrosis, NHL – non-Hodgkin lymphoma, MCL – mantle cell lymphoma, AML – acute myeloid leukemia, ALL – acute lymphoblastic leukemia, MDS – myelodysplastic neoplasm, DRI – Disease Risk Index,

### Transplant details

Except for one, all patients were transplanted from fully matched unrelated donors (MUD), using peripheral blood as the graft source. The conditioning regimen varied, tailored to each patient’s age, comorbidities, and disease risk index. Standard GvHD prophylaxis with CSA and a short course of MTX was administered to all patients receiving transplants from MUD. Anti-thymocyte globulin (ATG), specifically Thymoglobuline Sanofi™, was used for all these patients at a dose of 4.5 mg/kg. One patient, who received a transplant from a haploidentical family donor, was treated with post-transplant cyclophosphamide, followed by standard doses of CSA and MMF. All patients received standard prophylaxis for infections, which included oral levofloxacin (500 mg daily), intravenous acyclovir (5 mg/kg, thrice daily), and fluconazole (400 mg daily). Enoxaparin was administered daily in a prophylactic dose, adjusted according to the platelet count. Patients continued with their antihypertensive medications; only one patient was on a beta-blocker, at the lowest dosage.

### The management and treatment of bradycardia

All patients developed bradycardia within an hour of the start of CSA infusion, shortly after the infusion of HSCs, occurring within a week of the first CSA administration (median: 2nd day of immunosuppression; range: 1-4 days). In each case, sinus bradycardia without any additional arrhythmic disturbances was noted in the ECG recordings. The median minimal heart rate was 41 BPM (range: 35-50 BPM), and the median systolic and diastolic blood pressures were 92 mm Hg (range: 87-105 mm Hg) and 67 mm Hg (range: 56-81 mm Hg), respectively. Two patients presented with clinical symptoms of bradycardia, including atypical chest pain and syncope. Hypotonia was observed in three patients whose bradycardia lasted longer than 1 day. Neither neurological disorders nor myocardial infarctions were observed at the time of bradycardia. All patients remained in a stable condition. Of note, electrolyte levels in all cases remained within normal ranges, and CSA concentrations were between 100 and 160 ng/ml, slightly below the target therapeutic concentration. No troponin elevation was detected. Additionally, hypoglycemia was excluded in all patients. The exact electrocyte levels and CSA concentration are presented in Table 2.

**Table 2.** Details of the course of transplantation and bradycardia.

No.	Type of donor	Conditioning protocols^*^	TCI	HCT-CI	CSA start dosage [mg]	Day of CSA treatment when bradycardia occurred	Heart rate (HR) and ECG record at first episode of arrythmia	CSA concentration when bradycardia occurred [ng/mL]	Electrolyte levels when bradycardia occurred [mmol/l]	Duration of bradycardia [days]	Treatment of bradycardia
1.	MUD	FluBu3	RIC/RTC	2	260	2	HR = 35/min; sinus bradycardia	116	[Ca2+] = 7,8 [K+] = 4,3 [Mg2+] = 1,52 [Glc] = 80	3	CSA withdrawal, multi-electrolyte fluids infusion
2.	MUD	RituxFluTBI 4Gy	NMA	4	300	4	HR = 42/min; sinus bradycardia	131,8	[Ca2+] = 8,7 [K+] = 4,2 [Mg2+] = 2,03 [Glc] = 89	1	CSA withdrawal
3.	MUD	FluBu4	MAC	3	220	2	HR = 44/min; sinus bradycardia	122	[Ca2+] = 8,5 [K+] = 4,5 [Mg2+] = 2,03 [Glc] = 86	2	CSA withdrawal, multi-electrolyte fluids infusion
4.	Haploidentical	BFR	NMA	0	200	1	HR = 35/min; sinus bradycardia	108,3	[Ca2+] = 8,3 [K+] = 4,2 [Mg2+] = 1,71 [Glc] = 114	1	CSA withdrawal
5.	MUD	Cy/TBI 12 Gy	MAC	0	200	2	HR = 38/min; sinus bradycardia	127	[Ca2+] = 7,9 [K+] = 4,4 [Mg2+] = 1,6 [Glc] = 96	4	CSA and beta-blocker withdrawal, multi-electrolyte fluids infusion, atropine intravenously
6.	MUD	FluTBI	NMA	3	300	4	HR = 50/min; sinus bradycardia	156,6	[Ca2+] = 8,3 [K+] = 5,3 [Mg2+] = 1,66 [Glc] = 99	1	CSA withdrawal

Abbreviations: HSC – hematopoietic stem cells, TCI – transplant conditioning intensity, HCT-CI - Hematopoietic Cell Transplantation-specific Comorbidity Index, CSA – cyclosporine A, MUD – matched unrelated donor, SIB – sibling, FluBu3-4 – fludarabine and busulfan, RituxFluTBI – rituximab, fludarabine and total body irritation, BFR – bendamustine, fludarabine and rituximab, Cy/TBI – cyclophosphamide and total body irritation, FluTBI – fludarabine and total body irritation, RIC/RTC – reduced intensity conditioning/reduced toxicity conditioning, NMA – non-myeloablative conditioning, MAC – myeloablative conditioning

*All regimens and drug dosages were administrated according to the European Bone Marrow Transplantation (EMBT) recommendation

The median duration of bradycardia was 2.5 days (range: 1-4 days). The heart rate normalized promptly in all patients following CSA withdrawal, and only one patient required a one-time atropine injection due to prolonged and symptomatic arrythmia. Immunosuppressive therapy was continued using tacrolimus in all cases. There were no recurrent arrhythmic episodes during the hospital stay. The course of treatment and arrhythmia is presented in Table 2.

### Follow-up

All patients underwent full hematological recovery and were discharged from the transplantation department. The median follow-up was 37 months (range: 8-93 months) and, in spite of death of two patients, the overall survival remains 37 months (range: 16-79 months). None of the patients experienced further episodes of sinus bradycardia. Maintenance treatment with acalabrutinib was continued in two patients with mantle cell lymphoma.

Three patients experienced skin and gastrointestinal type of acute GvHD (aGvHD), successfully treated with medium dosages of steroids. Thereafter, two of them developed chronic GvHD (cGvHD). In one, liver, oral mucosa and conjunctiva were affected, whereas the second patient suffered from gastrointestinal disorders. Both patients received complex immunosuppressive therapy, including steroids, mycophenolic mofetil and extracorporeal photopheresis which allowed for symptom withdrawal and good cGvHD control.

Two patients died due to infectious complications within 7 and 2 months since allotransplantation (COVID-19 and sepsis, respectively). One patient with acute lymphoblastic leukemia (ALL) relapsed and underwent a second allotransplantation following successful salvage therapy of inotuzumab ozogamicin. She had received tacrolimus since the beginning of the second posttransplant immunosuppressive therapy and did not experience any arrythmia. As of the last follow-up on 10th January 2024, this patient remains in CR without symptoms of GvHD.

In three patients, the course was not complicated by aGvHD or cGvHD and they remained in complete remission (CR) until the last follow-up on 10 January 2024.

## DISCUSSION

Sinus bradycardia can be caused by various conditions, including cardiac disorders (e.g., ischemia, myocardial infarction and sick sinus syndrome) and non-cardiac factors (e.g., vasovagal reaction, hypothermia, hypoglycemia, hypothyroidism, increased intracranial pressure, electrolyte disorders), as well as drug intoxication (e.g., overdose of digoxin, beta-blockers).^7^ In the course of diagnosis, all these potential causes were excluded. The analyzed population is heterogenous, varying significantly in underlying diseases and conditioning. No gender predominance was observed. Although the arrythmia complication was mostly seen in patients over 50 years old, a case of a young patient in her twenties was also detected.

Despite the cardiovascular episodes and comorbidities in the medical histories of most patients, the only likely common cause of bradycardia was CSA administration, as evidenced by the rapid recovery of normal heart rate, monitored via ECG, following drug discontinuation in all patients. In each case, other causes of bradycardia, such as electrocyte and glucose imbalance, beta-blocker intoxication or atrioventricular block, were excluded. Furthermore, bradycardia occurred shortly after the beginning of CSA infusion. In four of the eight patients, immediate withdrawal of CSA was sufficient to restore a normal heart rate. In three other patients with prolonged bradycardia with subsequent hypotension, infusion of multi-electrocyte fluids and injection of atropine only allowed a temporary increase in heart rate. Eventually, permanent resolution of the arrhythmia was achieved after complete discontinuation of CSA.

Cardiovascular adverse effects of CSA have been predominately described as hypertension ,^8–10^ while arrythmia remains a rare phenomenon, often neglected in reports. Despite articles suggesting bradycardia as a result of multidrug interactions,^11–13^ the patients in our study did not receive the drugs mentioned in these reports. Contrary to the multidrug intoxication theory, Fujisaki G. et al. suggested that CSA itself could be the cause of bradycardia in a patient who underwent alloHSCT.^14^ However, the authors proposed a dosage-dependent cause of arrythmia, which was not observed in our study. A series of cases presented by Moulin D. et al. also indicated cyclosporine as a single agent causing side effects, including bradycardia.^15^ In their study, 74% of patients were reported with bradycardia, but it included a pediatric population after solid organ transplantation, which is not directly comparable due to significant differences in age and cyclosporine dosage.

Despite the limited reports of bradycardia associated with CSA in patients after alloHSCT, the supporting evidence for our research hypothesis can be found in animal studies. The modulation of baroreceptors by CSA, a factor in such adverse effects as hypotension, is well-documented in the literature.^8,16–20^ These receptors also play a crucial role in arrhythmic disorders. Although there is attenuation of arterial baroreceptor effects in increasing heart rate,^16^ the baroreflex-mediated response is variable, and does not necessarily manifest as tachycardia. The heart rate response to such modulation depends on chronotropic responses, reflecting the relative contributions of vagal and sympathetic components.^21–23^ The two autonomic efferents contribute differently to CSA-induced impairment of reflex chronotropic responses to impair baroreceptors. While attenuation of both vagal and sympathetic signals can lead to bradycardia due to the depressant effect of CSA on reflex tachycardia, the impairment of reflex bradycardia by CSA involves a preferential attenuation of vagal activity.^24^

The exact mechanism by which CSA modifies autonomic neurotransmission remains to be fully understood and warrants further investigation. It is unclear whether CSA directly interacts with specific vagal and sympathetic control sites, or if the alterations in heart rate are secondary effects of hemodynamic changes. Additionally, the potential direct impact of CSA on cardiac tissue should not be overlooked. The main target of CSA is the release of calcium (Ca2+) via Ryanodine receptors (RyR). This sensitization of RyR results in increased Ca2+ release, which leads to a reduction of sarcoplasmic reticulum Ca2+ content. This, combined with the passive Ca2+ leak also induced by CSA, can result in a sustained decrease of Ca2+ release and, consequently, a reduction in heart rate.^25^

It should be noted that all patients had pre-existing cardiovascular disorders, which were not a contraindication to allotransplantation, but may have made patients particularly susceptible to cardiovascular disorders caused by CSA administration. Identification of specific risk factors, however, requires further comprehensive prospective studies to allow detailed comparative statistical analysis. Nevertheless, we advocate close monitoring, including continuous ECG recording, of patients at high cardiovascular risk during the first days of CSA infusion, including especially on day +1 after alloHSCT. If sinus bradycardia occurs, CSA infusion should be stopped. Once other potential causes of arrhythmia have been ruled out, it is recommended to change the immunosuppressive drug to tacrolimus.

Finally, it is worth mentioning that, after changing immunosuppressive treatment from CSA to tacrolimus, another calcineurin inhibitor, no further episodes of bradycardia were observed in the patients included in our study. This suggests a CSA-specific mechanism of action that differs from its well-described immunosuppressive effect. Therefore, despite its long history of clinical use, CSA still requires further research to fully understand its complex effects on human physiology.

## CONCLUSION

Considering the limitations of the study, such as retrospective analysis, a small number of cases, and heterogeneity within the research group, sinus bradycardia remains the most probable adverse drug effect of cyclosporine A in the presented case series. The patients varied significantly in age, diagnosis, and treatment, with the only observed common factor being CSA administration. This hypothesis is further supported by the fact that bradycardia occurred early in the CSA treatment period and, following CSA withdrawal, heart rate normalized quickly in all patients. While there is a likelihood of pre-existing cardiovascular disorders playing a role in the risk of CSA-induced bradycardia, further studies with larger research groups are required to confirm this. Nevertheless, the continuation of immunosuppressive therapy with another calcineurin inhibitor, tacrolimus, has been safe and free of arrhythmic episodes. It is worth emphasizing that, given the limited information on bradycardia during CSA treatment, our single-center experience may be helpful in procuring prompt diagnosis and therapeutic intervention in daily clinical practice. A better understanding of CSA interactions and its potential adverse effects can lead to more accurate medication administration, and identification of patients at higher risk of serious drug complications.

### Conflict of Interest

No conflict of interest was declared by the authors.
